# Adverse health and environmental outcomes of sewage treatment plant on surrounding groundwater with emphasis on some mitigation recommendations

**DOI:** 10.1007/s10653-022-01413-7

**Published:** 2022-10-28

**Authors:** A. T. Kandil, K. Haggag, A. A. Gamal, M. G. Abd El-Nasser, W. M. Mostafa

**Affiliations:** 1 Chemistry Department, Faculty of Science, Helwan University, Helwan, Egypt; 2 Egyptian Atomic Energy Authority, Cairo, Egypt; 3 Academy of Scientific Research and Technology (ASRT), Cairo, Egypt

**Keywords:** Sewage plant effect, Water quality, Health risk exposure, Trace element speciation, Mitigation of contamination

## Abstract

Water quality deterioration hinders economic and social development in developing countries that are facing freshwater security and shortages. Based on the collection of 29 water samples, this study focused on the relationship between sewage treatment plant and groundwater system surrounding it using multidisciplinary approach that combines the characterization of groundwater system and its connection with surrounding canal and drains, using chemical and isotopic characterization revealing that there is a direct relation between the surface water system and surrounding groundwater system. About 58% of the groundwater samples and all surface water samples in the investigated area are threatened by high concentrations of trace elements. The multivariate statistical analysis elucidates that anthropogenic effect and fertilizers sewage contamination are the main causes of groundwater pollution. Nearly, 31% and 11.5% of groundwater samples were posing oral chronic non-carcinogenic health risk and dermal chronic risk for adult, respectively, while all surface water samples were posing oral chronic non-carcinogenic health risk, with no dermal hazard. The uncharged species of Fe and Al are expected to be more mobile in groundwater because they would not be attracted to the surface charge of minerals. Inorganic ligands (HCO_3_^−^, SO_4_^2−^, Cl^−^, and NO_3_^−^) act as nucleation centers that were linked with those trace elements creating new species with higher solubility degree in water that are transported away randomly for long distances in the water path.

## Introduction

Deterioration of water quality is considered as a major challenge that hinders economic and social development in developing countries where freshwater security and shortages are problematic issues. Raising water demands and the rapid and continuous increment of population together with the climatic change put stresses on freshwater resources quality and quantity. Human health risk assessment of groundwater is mandatory and essential issue that correlates and quantifies the environmental pollution loads on human being. This can be done through the estimation of potential risk sources indices, by estimating the values of risk indices, in addition to determining the health consequences of exposure.

Although water is considered to be a survival factor for human life-sustaining and its related ecosystem, every year more than 3.4 million people around the world die suffering from diseases originated from using polluted water (WHO, 2017**).** Recently, many studies discussed the sources of groundwater pollution and their influences on ecosystems components. They revealed that the pollution of the ecosystem by heavy metals harms the living organisms and human health (Aguirre et al., 2019; Lupi et al., 2019). The geochemical characteristics of the water system, ecotoxicology of the heavy metals, and high pollution level related play a vital role in controlling the stability/mobility and availability that affects the pathways of trace metals in water bodies (Ali et al., 2016; Zhao et al., 2021). Hence, the full understanding and explanation of the source, fate, and potential effects of these parameters are the main factor in environmental protection management as it might give more details on systematically alteration monitoring process, and risk assessment to preserve this valuable ecological component and avoiding any potential risk for human water related diseases and infections(Mirzaei et al., 2020).

Chemical species of trace elements have a direct effect on their environmental chemistry concerning their mobility, potential toxicity to living organism. The geochemistry of trace metals is strongly influenced by its speciation. As it exhibits a variety of aqueous and particulate species through the association with some ligands such as Cl^−^,OH^−^, SO_4_^2−^,F^−^, and HCO_3_^−^to form complexes.

The sewage treatment plant especially in developing countries might add some constrains and threats on the surrounding environmental component. A special focus on Cairo city as the increment in the population in this capital increases the risk of groundwater quality deterioration. The combined effects of the applications of fertilizer, pesticides, in addition to, the use sewage water for irrigation, which might have a diverse impact on groundwater quality in this area might lead to serious health problems for its residents.

### Aim of the work

The present work aimed to characterize the groundwater resources in Shoubra area and determination of its quality in the vicinity of sewage treatment plant in the study area. In this paper, several combined tools have been used to achieve this aim; hydrochemistry, environmental stable isotopes, and microbiology of collected samples were integrated with statistical analysis for complete characterization of the groundwater system and its realtion with surface water, in addition to that geochemical modeling has been functioned as the major ions and trace elements levels and their speciation in the groundwater, as it would be beneficial prior to remediation and mitigation actions. This might help in water resources protection and management issues, through the recognition of the metals pollution trends and its sources in water their influence on environmental systems.

### Study area

The study area, Shoubra area, is characterized by a lot of industrial activities considered one of the main four big cities in Egypt. It is located in Qalyubia governorate is in the Nile Delta, north of Cairo, and extended between latitudes 30°07′30″ to 30°32′92″ N and longitudes 31°3′30″ to 31°34′30″ E, with a total area of 270 km^2^ and a population of 1,073,000.

**Fig. 1:**
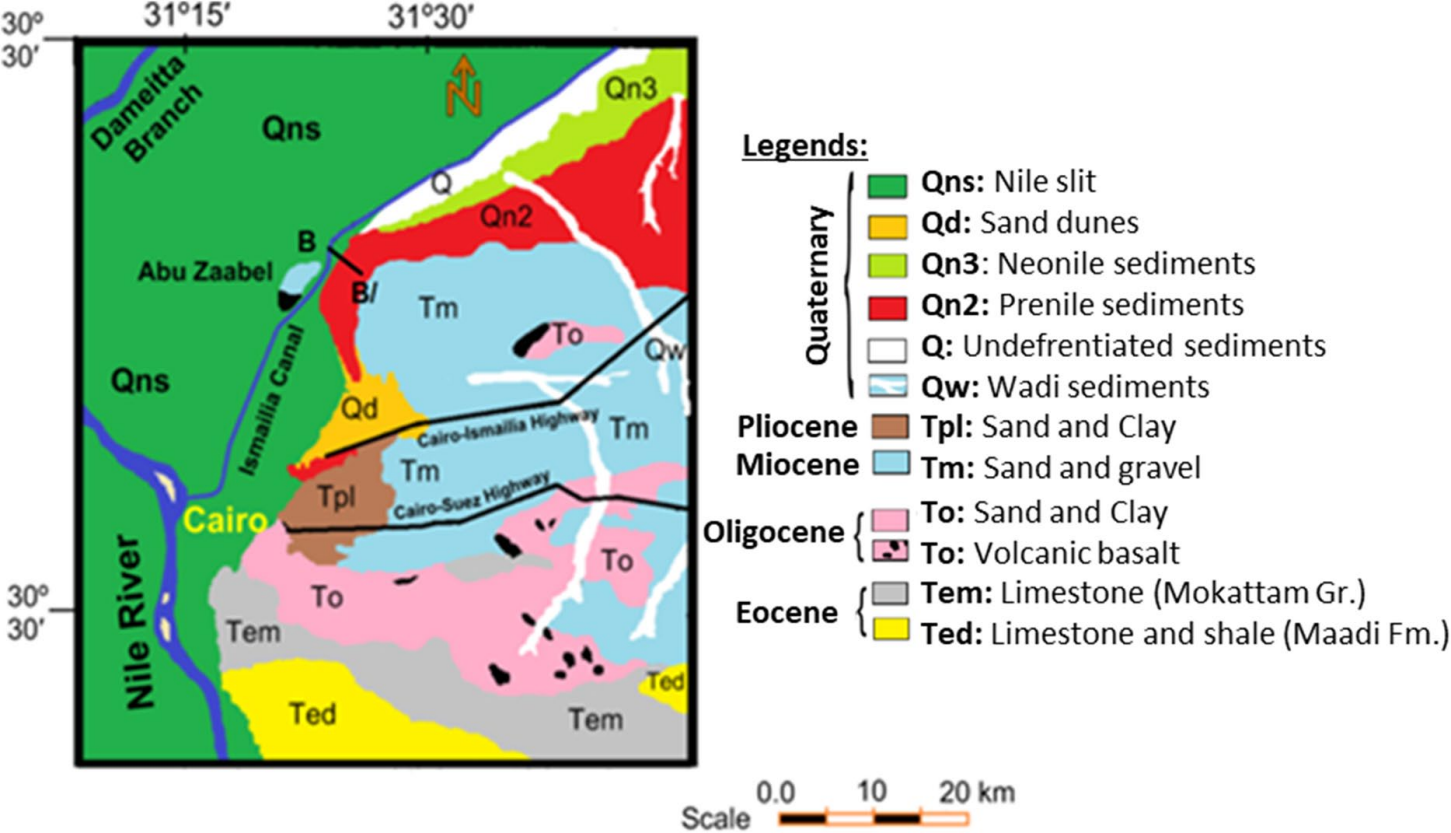
Geologic map of the study area and its vicinities **(**CONOCO, 1987**)**

The study area is dominated by Holocene Nile deposits consisting of Nile silt, sand, and sandy clay deposits, with thickness ranging from 0 to 20 m. It is underlain by the Pleistocene sediments which are formed mainly of sand and gravels with intercalations of clay lenses. These sediments have a variable thickness at the eastern parts of the Nile Delta, ranging from zero in the south to nearly 600 m, while at the northwestern parts they may reach 900 m (Khalil et al., 1988; Mansour, 2020). To the east of the area, the oldest Tertiary rocks (Pliocene, Miocene, Oligocene, and Eocene sediments) appear on the surface. The Oligocene basalt appears on the surface as small exposure in the southern parts of the study area at Abu Zaabal region shown as Fig. 1. The Oligocene rocks are dominated by sands, gravels, and fractured basalt. The thickness of these rocks varies from 30 to 100 m and acts as a deep aquifer below the basaltic sheets. Capping basalt may (about 30 m) overlie the fluviatile sands and gravels. The Miocene sediments are present in some places of the study area and composed of alternating sandy limestone and sandy marls of shallow marine origin. Faulting is the dominating structural element and is mainly vertical and facilitates both the downward passage of water and up flow from deep aquifers (Elawadi et al., 2006; Farag & Ismail, 1955; Sultan et al., 2008).

### Hydrogeology

There are two main aquifers in the study area, Quaternary and Miocene aquifer. The Quaternary aquifer is the main bearing formation in the study area formed of heterogeneous materials of yellowish brown, medium to coarse-grained sands with occasional soft and brown clay lenses and few amounts of gravels and calcareous materials Fig. 1. It has a variable thickness at the eastern parts of the Nile Delta, ranging from zero in the south to nearly 600 m, while at the northwestern parts the thickness may reach 900 m (Khalil et al., 1988; Mansour, 2020). The Quaternary aquifer is overlain by a semi-permeable Nile silty layer (aquitard) of Holocene age, rendering the aquifer under semi-confining conditions. The Nile silt layer is composed of heterogeneous and anisotropic materials (silt, clay, and sand). It has a thickness that ranges from 15 m adjacent to Ismailia Canal due east to 5 m in the western part of the study area. The depth of water below the land surface is shallow. The groundwater levels range from about 12.8 m above mean sea level (MSL) under the cultivated lands to more than 14.9 m (MSL) under the westwards and 16.3 m (MSL) southwestwards in the urban areas, (Farid, 1985; Nofal et al., 2015; RIGW, 1989; Sherif et al., 2012; Taha et al., 1997; Yehia, 2000). The groundwater approximately moves from all directions toward the cultivated lands. The Miocene sediments, which are made of overlapping sandy dolomite and sandy marls of deep marine origin, may be found in several areas of the study area. The Ismailia freshwater canal, which is located on the southern outskirts of the Eastern region of Nile Delta and has a higher level of water than groundwater, is the principal source of groundwater refill in the Eastern region of Nile Delta. (Nofal et al., 2015).

## Materials and methods

For this study, 29 water samples were collected from Shoubra area, complete physico-chemical analyses of the collected water samples were conducted according to the Standards Methods for Examination of Water and Wastewater. The pH was measured at 25°C in the field using pH meter, (Model: Cole Parmer date meter). Electrical Conductivity (EC) was measured in the field using EC meter (Model: Cole Parmer date meter CON 410 series). Major ions (Na^+^, K^+^, Ca^2+^, Mg^2+^, HCO_3_^−^, Cl^−^ and SO_4_^2−^) as well as trace elements(Al, Ba, Fe, Mn, Sr, Zn, Cr, V)were measured using ICP (Inductive Coupled Plasma) at Egyptian Desert Research Center. The stable isotopes (Oxygen -18 and Deuterium) were measured according to the standard methods using Laser spectroscopy Piccaro (Model 2120i) in the Central laboratory of Isotope Hydrology in Egypt. Microbiological analyses(Total Coliform, Fecal Coliform, Salmonella and Shigella, and Total Count)were carried out according to APHA (2017) at Agricultural Research Center (ARC).

Suitability of groundwater for human uses mainly depends on groundwater geochemistry; hence, each groundwater system has its unique chemical composition that might be altered as a result of many factors. Recently, a new technique adapted that combines several effects of distinct water quality parameters on the whole quality of water at certain location and time known as water quality index (Canadian Environmental Quality Guidelines, 2001). This index (WQI) is characterized by lower score indicating better water quality, and higher score indicating worse water quality, providing a rapid unique score of groundwater status for non-specialized decision-maker, Table 1.

**Table 1:** CCMEWQI method categorization (Canadian environmental quality guidelines, 2001)

Categorization	Index value
Excellent	95–100
Good	80–94
Fair	65–79
Marginal	45–64
Poor	0–44
Unsuitable	Detection of microorganisms

The Canadian water quality index (Rocchini & Swain, 1995) that consists of three factors (Scope F1, frequency F2, and amplitude F3) was utilized in this study according to the method adopted by Canadian Environmental Quality Guidelines, 2001. Fourteen hydrochemical parameters were used as variables from the data set of hydrochemical analyses for all samples of groundwater and surface water. Once the variables and the objectives have been defined, three factors (F1, F2, and F3) for each sample were used to calculate the water quality Index (CCME WQI). The calculation of each factor and the overall quality index described as follows.

F1 (Scope) represents the percentage of variables that do not meet their objectives at least once during the time period under consideration (“failed variables”), relative to the total number of variables measured for only one sample Eq. 1:

1 $$F1 = \frac{{{\text{Number}}\;{\text{of}}\;{\text{Failed}}\;{\text{Variables}}\;{\text{for}}\;{\text{one}}\;{\text{sample}}}}{{{\text{Total}}\;{\text{Number}}\;{\text{of}}\;{\text{Variables}}}} \times 100$$

F2 (Frequency) represents the percentage of individual tests that do not meet objectives (“failed tests”) and can be calculated as follows Eq. 2:

2 $$F2 = \frac{{{\text{Number of}}\;{\text{Failed Variables}}\;{\text{for}}\;{\text{all}}\;{\text{samples}}}}{{{\text{Total}}\;{\text{Number}}\;{\text{of}}\;{\text{Variables}} \times {\text{Number}}\;{\text{of}}\;{\text{samples}}}} \times 100$$

F3 (Amplitude) represents the amount by which failed test values do not meet their objectives. F3 is calculated in three steps.

i) The number of times by which an individual concentration is greater than (or less than, when the objective is a minimum) the objective is termed an “excursion” and is expressed as follows in Eq. 3 (When the test value must not exceed the objective):

3 $${\text{Excrusion}}_{i} = \left\{ {\frac{{{\text{Failed}}\;{\text{Test}}\;{\text{Value}}_{i} }}{{{\text{Variables}}_{j} }}} \right\} - 1$$

ii) The collective amount by which individual tests are out of compliance is calculated by summing the excursions of individual tests from their objectives and dividing by the total number of tests (both those meeting objectives and those not meeting objectives). This variable, referred to as the normalized sum of excursions, or nse, is calculated as in**:**Eq. 4

4 $${\text{nse}} = \sum\limits_{{i = 1}}^{n} {\frac{{{\text{Excursion}}_{i} }}{{{\text{number}}\;{\text{of}}\;{\text{Tests}}}}}$$

iii) F3 is then calculated by an asymptotic function that scales the normalized sum of the excursions from objectives (nse) to yield a range between 0 and 100, Eq. 5:

5 $$F3 = \left\{ {\frac{{{\text{nse}}}}{{0.01{\text{nse}} + 0.01}}} \right\}$$

Once the F1, F2 and F3 factors have been determined, the overall water quality index CCMEWQI can be calculated by summing the three factors as if they were vectors according to the following equation Eq. 6.

6 $${\text{CCMEWQI}} = 100 - \left\{ {\frac{{\sqrt {F1^{2} + F2^{2} + F3^{2} } }}{1.732}} \right\}$$

## 3. Results and discussion

### 3.1 Isotopic characterization of groundwater samples

Oxygen-18 (^18^O) and Deuterium (D) are commonly used as natural tracers in hydrology for elucidation groundwater recharge sources and its flow paths, as well as, determining the hydrological processes that affecting the chemical reactions, evaporation and mixing processes that occur along hydrological pathways. The isotopic composition of collected groundwater and surface water samples from Canal is statistically described in Table 2; it was found that *δ*^18^O values range from -0.75 to 4.52‰, and *δ*D values range from 2.59 to 32.96‰, respectively.

**Table 2:** Descriptive static of the stable isotopes in the study area

Item	*δ*^18^O(‰)	*δ*D(‰)	T (TU)
St. Error	0.27	1.61	0.67
Median	2.89	23.50	1.96
St. Dev	1.39	8.3	1.77
Variance	1.94	69.64	3.14
Kurtosis	0.44	0.32	-2.13
Skewness	-1.10	-1.03	-0.13
Range	5.27	30.37	4.40
Min	-0.75	2.59	B.D.L
Max	4.52	32.96	3.68
Mean	2.49	20.97	1.43
B.D.L.: Below Detection Limit			

**Fig. 2:**
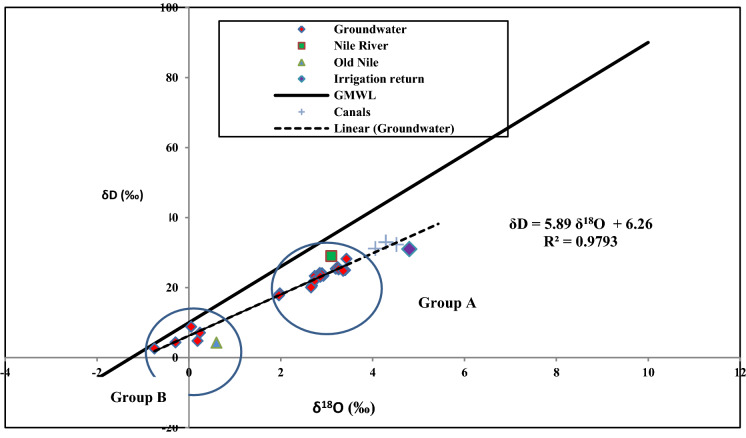
The diagram of *δ*^18^O (‰) and *δ*D (‰)

The diagram of *δ* ^18^O and *δ*D is plotted in Fig. 2, with three possible end members that might contribute the aquifer recharge are illustrated in, Recent Nile (*δ*D = 29‰ and *δ*^18^O = 3.1‰), Old Nile Before High Dam Construction (*δ*D = 4.3‰ and *δ*^18^O = 0.6‰) and return Irrigation Water (*δ*D = 31‰ and *δ*^18^O = 4.8‰). The regression line for *δ*^18^O and *δ*D has an equation that is defined by (*δ*D = 5.89 *δ*^18^O + 6.26), the location of the samples below Global Meteoric Water Nile with slope of 5.89 revealing evaporation process before infiltration in less permeable zones as a results of the existence of silt component in the aquifer matrices. The plotted canal samples were isotopically enriched more than Recent Nile indicating more evaporation occurring in canals. All ground water samples fall between recent Nile component (represented by Ismailia El-Sharkaweya Canals) and old Nile. Groundwater samples are discretized into two main groups, group (A) that is mainly recharged from Recent Nile component that comprises about 80% of the samples; group (B) comprises 20% of the collected samples and mainly recharged from Old Nile buried channels in the study area, revealing that the Old Nile component and Recent Nile are two main components of recharge in this localities. The majority of samples (80%) are recharged mainly from Nile system (canals and drains).

**Fig. 3:**
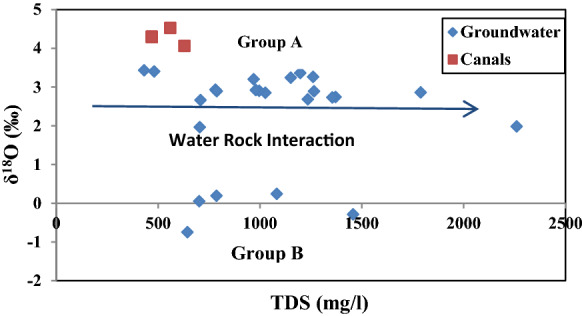
Diagram of *δ*.^18^O (‰) vs TDS (mg/l)

The relationship between *δ*^18^O and TDS have been plotted to give more declaration about the geochemical mechanism of groundwater caused by the evaporation or water–aquifer matrices interaction. The same groups appear in Fig. 3, and both groups show a limited increment in the isotopic contents accompanied by wide observed increment in TDS values indicating water–rock interaction between feeding water and aquifer matrices.

**Fig. 4:**
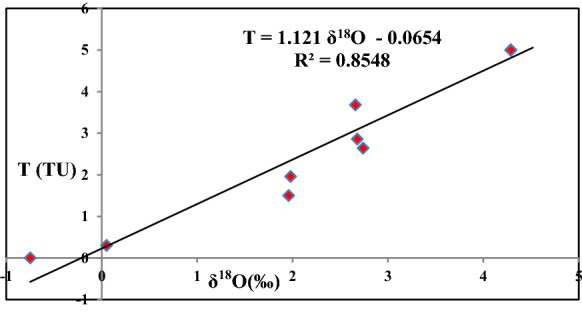
Diagram of Tritium (TU) vs *δ*.^18^O (‰)

The results of tritium analysis conducted on a selected number of groundwater samples in the study area are summarized and shown in \* MERGEFORMAT Table 2. The tritium content of only one sample is either undetectable or varying in the range of the lower limit of detection confirming the presence of paleo-recharge source of more than 45 years; no upper limits on this age can be estimated from the tritium data of this sample revealing the contribution of old Nile buried branches effect. On the other hand, the rest of the analyzed samples have tritium content in the range from (0.1TU to 4.55 TU), suggesting a contribution from recently infiltrated Nile water (adjacent Ismailia and El-Sharkaweya Canals) to this groundwater from recently infiltrated Nile water that seeps to adjacent Ismailia and El-Sharkaweya Canals. The relationship between tritium content in groundwater against *δ*^18^O is shown as Fig. 4 with proportional trend (Tritium = 1.121 *δ*^18^O-0.0654) indicating the effect of recent Nile contribution through Ismailia and El-Sharkaweya Canals to the aquifer.

### 3.2 Water quality assessment

Nearly about 23% of the groundwater samples were ranged in categorization of fair water quality (occasionally threatened), while it was 42% as good water quality revealing that they have a minor degree of threat; the rest of samples (35%) ranged from marginal to unsuitable revealing direct continuous threaten. In case of surface water, 67% showed good water quality around Ismailia and El-Sharkaweya canals, while the rest 33% was marginal; Table 3.

**Table 3:** Water quality index of the study area's samples

	Sample(s)	WQI	Microorganisms				WQI Class
Type			Total coliform	Fecal coliform	Salmonella and shigella	WQI for MICRO	
Ground water samples	1	89.371	9	7	4	Unsuitable	Unsuitable
	2	77.545	Nd	Nd	Nd	-	Fair
	3	84.745	Nd	Nd	Nd	-	Good
	4	81.320	Nd	Nd	Nd	-	Good
	5	87.295	9	7	5	Unsuitable	Unsuitable
	6	90.191	Nd	Nd	Nd	-	Good
	7	89.443	Nd	Nd	Nd	-	Good
	8	88.814	24	20	15	Unsuitable	Unsuitable
	9	89.423	10	8	6	Unsuitable	Unsuitable
	10	71.961	Nd	Nd	Nd	-	Fair
	11	47.875	Nd	Nd	Nd	-	Marginal
	12	90.191	Nd	Nd	Nd	-	Good
	13	80.905	Nd	Nd	Nd	-	Good
	14	86.530	13	11	8	Unsuitable	Unsuitable
	15	85.329	17	14	12	Unsuitable	Unsuitable
	16	89.305	Nd	Nd	Nd	-	Good
	17	88.936	25	23	Nd	Unsuitable	Unsuitable
	18	84.175	Nd	Nd	Nd	-	Good
	19	90.191	Nd	Nd	Nd	-	Good
	20	74.216	Nd	Nd	Nd	-	Fair
	21	77.032	28	25	Nd	Unsuitable	Unsuitable
	22	74.904	Nd	Nd	Nd	-	Fair
	23	77.882	Nd	Nd	Nd	-	Fair
	24	83.374	Nd	Nd	Nd	-	Good
	25	69.956	Nd	Nd	Nd	-	Fair
	26	84.237	Nd	Nd	Nd	-	Good
Surface water samples	27	88.160	Nd	Nd	Nd	-	Good
	28	77.184	Nd	Nd	Nd	-	Good
	29	60.226	Nd	Nd	Nd	-	Marginal

### 3.3 Causes of water pollution using statistical approach

**Fig. 5:**
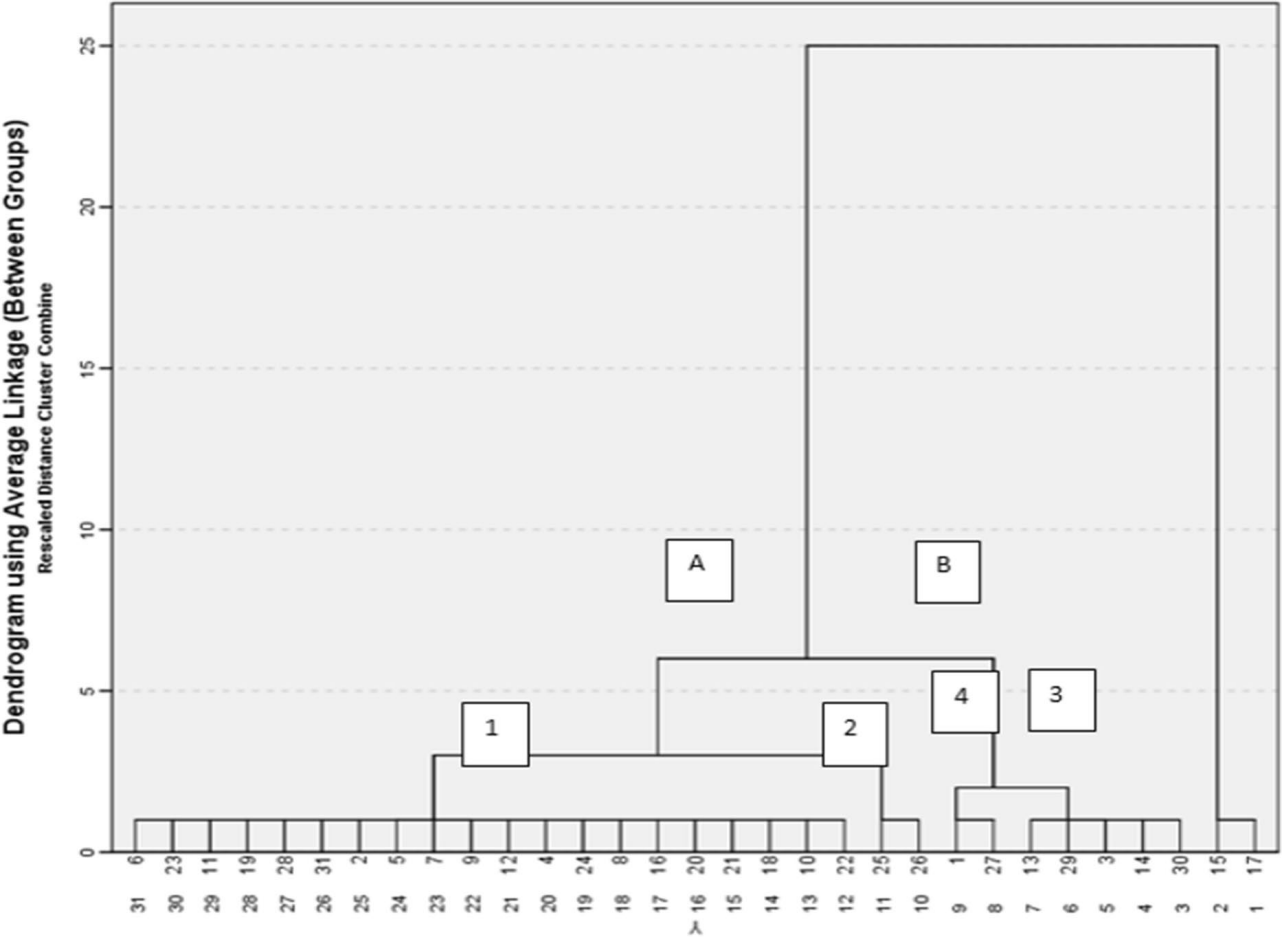
Dendrogram analysis of the collected groundwater and surface water samples

The multivariate statistical analysis has been utilized in this study as a trial to correlate and relate the variables for comparing them within the whole samples domain to elucidate the causes of groundwater pollution in the studied area. An attempt has been done by choosing the most threatened variable (physicochemical variables as: Hardness, pH, TDS; and chemical variables as: SO_4_, NO_3_, Al, Ba, Fe, Mn, Sr; in addition to microbial variables as: Total Coliform, Fecal Coliform, Salmonella and Shigella, Total Count) to groundwater quality in the study area. Hierarchical cluster Analysis (HCA) and Principal Components Analysis (PCA) using SPSS.22 statistical software have been used for fourteen variables chosen from the measured items in the study to trace and detect the pollution sources in groundwater, through classifying them into groups and components representing the features and variations. The hierarchical cluster analysis (HCA) classified the water samples into two groups, based on the combination of interval based on multiple parameters from different samples and according to their similarity to each other. The dendrogram, shown as Fig. 5, classified the majority of samples (88%) according to the proximity of the water quality parameters into two major groups (A: 76% and B: 24%). Group (A) comprises two clusters: cluster (1) where majority of groundwater samples are included (59%), and cluster (2) comprises 7% of the samples (one groundwater sample and one surface water). On the other hand, group B includes two clusters: cluster (3) that included only 7% of the samples (one groundwater sample and one surface water sample), and cluster (4) for about 17% of samples including both groundwater samples and surface water samples. Principal Components Analysis (PCA) technique was used as it has the large data reduction ability for the fourteen variables into a set of variables called Principal Components, based on their variances and eigenvalues relations shown in Table 4. Five components representing about 82.8% of the samples (Eigen values > 1) were considered to be representative for the overall characteristics of the water quality data set. The first principal component accounts for 36% of the variance in the data set and combines only chemical and trace elements variables Hardness, TDS, SO_4_,NO_3_, Al, Sr, Fe, and Ba; the presence of trace elements and nitrate in this group reflecting anthropogenic effect and application of fertilizers.

**Table 4:** Principal component analysis of the collected groundwater and surface water samples

Total variance explained									
Component	Initial eigenvalues			Extraction sums of squared loadings			Rotation sums of squared loadings		
	Total	% Of Variance	Cumulative %	Total	% Of variance	Cumulative %	Total	% Of Variance	Cumulative %
1	5.050	36.075	36.075	5.050	36.075	36.075	3.710	26.498	26.498
2	2.482	17.729	53.804	2.482	17.729	53.804	2.871	20.508	47.006
3	1.599	11.421	65.225	1.599	11.421	65.225	2.270	16.211	63.217
4	1.381	9.868	75.093	1.381	9.868	75.093	1.389	9.924	73.141
5	1.085	7.752	82.845	1.085	7.752	82.845	1.359	9.704	82.845
6	.769	5.491	88.336						
7	.563	4.019	92.355						
8	.395	2.819	95.174						
9	.305	2.176	97.350						
10	.175	1.253	98.603						
11	.106	.759	99.362						
12	.067	.477	99.839						
13	.022	.155	99.994						
14	.001	.006	100.000						
Component Matrix using Principal Component Analysis									
	Component								
	1	2	3	4	5				
Hardness	.920	.240	.070	.095	.010				
pH	− .262	− .413	− .017	− .278	.755				
TDS	.915	.276	-.051	-.033	-.023				
SO_4_	.829	.138	.027	-.296	.156				
NO_3_	.483	.166	.562	-.364	.366				
Al	-.017	-.193	.129	.719	.466				
Ba	.412	.158	.615	.459	.015				
Fe	.525	.144	-.732	.264	.166				
Mn	.609	.167	-.554	.032	.212				
Sr	.875	.249	.164	.003	-.140				
Total Coliform	− .511	.815	− .045	− .082	.134				
Fecal Coliform	− .512	.807	− .054	− .081	.137				
Salmonella and Shigella	− .410	.715	.050	− .079	.084				
Total Count	− .322	.362	.060	.506	.047				

The second component represents about 17.7% of the variance and combines Microbial contamination variables (Total Coliform, Fecal Coliform, Salmonella and Shigella, and Total Count) indicating a direct contamination from sewage plant to the surrounding groundwater. On the other hand, Ba and NO_3_ combine in the third group with variance 11.4% with negative correlation and association and combination of Fe and Mn, revealing that this samples might be affected by fertilizers. The fourth group of variances 9.8% includes Ba and Al with negative association of microbial contamination variables indicating industrial pollution or dissolution of aquifer matrices rather than sewage pollution. Finally, the fifth group with variance 7.8% is influenced only by pH with some association of Al.

### 3.4 Health risk exposure assessment

In this study, the assessment of the human health risks of groundwater is used to correlate and link environmental contamination with human health (Çelebi et al., 2014; Zhang et al., 2019; Tirkey et al., 2017; Singh et al., 2018). The potential risk sources indices have been determined by estimating the values of risk indices then calculated and estimating the health consequences of exposure for adults via both the direct ingestion of the water and dermal contact using the method applied in EPA (Hagagg et al., 2020; Ji et al., 2020; Li et al., 2017). Chronic non-carcinogenic health risk for adults via exposure of both oral and dermal routes is summarized in Table 5 for groundwater samples. In case of oral exposure, nearly about 31% of groundwater samples and 11.5% of samples were imposing oral and dermal chronic risk for adult (> 1), respectively. On the other hand, all surface water samples were posing oral Chronic non-carcinogenic health risk, with no dermal hazard.

**Table 5:** Statistical analyses of the calculated of the oral and dermal non-carcinogenic hazard quotients for groundwater samples

Item	ORAL HQ	Dermal HQ	All risks
St. Dev	2.35	0.63	2.97
Variance	5.51	0.39	8.85
Kurtosis	12.41	12.41	12.41
Skewness	3.3	3.3	3.3
Range	11.09	2.97	14.06
Min	0.03	0.01	0.04
Max	11.12	2.97	14.1
Mean	1.34	0.36	1.69

### Geochemical modeling and its role in mitigation actions

The correlation between TDS and Sr, Mn, Fe, Al, Ba, and Cr has been calculated to be 0.78, 0.61, 0.57, -0.02, 0.30, and 0.33, respectively. Sr,Mnand Feshowed a positive higher correlations with TDS concentrations revealing that Sr interacts with mineral surfaces through relatively weak outer-sphere coordination (McBride, 1997), while Mn and Fe adsorption could beoccur through outer- and inner-sphere coordination (Goldberg, 2005; Peak et al., 2003).This might explain the lower correlation between them and TDS as compared to that of Sr. On the other hand, concentrations of Al, Ba, Cr were not/ only slightly, affected by TDS as those elements seem to interact with mineral surfaces through inner-sphere coordination.

The positive correlation between TDS and Trace elements indicates the continuous dissolution of aquifer matrices minerals and amorphous phases; hence, this mineral dissolution releases trace elements to the aqueous phase accompanied by increasing the TDS concentration of groundwater. Visual MINTAQ geochemical modeling program was used in this work for calculating the distribution of groundwater aqueous species and the saturation degree of groundwater relative aquifer matrices for each sample to indicate their effect on the distribution of trace element in groundwater. Table 6 confirms the assumption of continuous dissolution of minerals (under-saturation) within the aquifer matrices, Gibbsite and Barite that was supersaturated.

**Table 6:** Saturation indices results based on MINTAQ geochemical model

Item	Mean	Standard error	Median	Standard deviation	Sample variance	Kurtosis	Skewness	Minimum	Maximum
Calcite CaCO_3_	− 0.58	0.07	− 0.63	0.35	0.12	3.05	1.47	− 1.05	0.52
Dolomite CaMg(CO_3_)_2_	− 0.27	0.15	− 0.31	0.75	0.56	3.50	1.26	− 1.62	2.15
Smithsonite ZnCO_3_	− 3.35	0.18	− 3.51	0.77	0.60	2.10	0.87	− 4.61	− 1.27
Strontianite SrCO_3_	− 2.12	0.07	− 2.19	0.34	0.12	6.06	1.88	− 2.65	− 0.88
Witherite BaCO_3_	− 3.05	0.08	− 3.10	0.40	0.16	2.45	1.39	− 3.66	− 1.87
Siderite FeCO_3_	− 1.30	0.15	− 1.34	0.79	0.62	1.19	0.05	− 3.18	0.59
Rhodochrosite MnCO_3_	− 1.14	0.16	− 1.08	0.81	0.66	− 0.44	0.06	− 2.62	0.58
Anhydrite CaSO_4_	− 1.76	0.06	− 1.76	0.29	0.08	− 0.50	0.20	− 2.28	− 1.19
Gypsum CaSO_4_	− 1.46	0.06	− 1.46	0.29	0.08	− 0.43	0.21	− 1.98	− 0.88
Barite BaSO_4_	1.29	0.07	1.34	0.37	0.13	0.99	− 0.95	0.30	1.85
Celestite SrSO_4_	− 1.97	0.06	− 1.96	0.30	0.09	− 0.18	− 0.21	− 2.55	− 1.37
Halite NaCl	− 6.66	0.09	− 6.63	0.46	0.21	− 0.11	− 0.43	− 7.63	− 5.81
Gibbsite Al(OH)_3_	2.86	0.10	2.81	0.43	0.18	− 0.02	0.14	2.05	3.70

Trace elements in solution mainly exist as complexes with halides, sulfate, phosphate, hydroxides, carbonates, DOC, or as free ions. Chemical speciation shows the distribution of a chemical element between different molecular ionic forms in water (Apostoli, 2006; Zhao et al., 2021).

By modeling, the chemical species of these elements will demarcate the behavior of these elements in the surrounding environment. This would give more insights about their mobility and their bioavailability to living organism, which might help in determining their potential toxicity.

Metal speciation studies for the samples were characterized, Table 7, to determine the un-complexed form of Ba, Mn, Zn, and Sr existed as free radical ion in the hazardous samples and were ranged from 85.74 to 96.5%, 55.41% to 80.7%, 75.59% to 91.5%, and 79.88 to 91.5%, respectively. It suggests that those elements would be available for further sorption process and can be mitigated through sorption processes. The mobility of those elements will not increase in our case as results of absence of acidic conditions and low dissolved organic matter content. A small extent of these elements existed as inorganic ligands. Nevertheless, the uncharged species of Fe and Al are expected to be more mobile in groundwater because they would not be attracted to the surface charge of minerals. Inorganic ligands (HCO_3_^−^, SO_4_^2−^, Cl^−^, and NO_3_^−^) act as nucleation centers that were attached and linked with those trace elements creating new species with higher solubility degree in water that are transported away randomly for long distances in the water path. Predominant ionic species of Fe and Al in groundwater samples were exist as metal hydroxides and they might show toxic effects in their inorganic forms.

**Table 7:** Speciation percentage of Trace elements for contaminated groundwater samples in the study area

Item	Mean	St.Dev	Variance	Kurtosis	Skewness	Min	Max	Count
AlOH^2+^	0.06	0.08	0.01	3.34	1.83	0.01	0.19	4
Al(OH)^2+^	0.79	1.12	1.25	12.40	3.41	0.20	4.86	17
Al(OH)_3_ (aq)	5.55	2.68	7.17	7.26	2.50	3.30	14.32	17
Al(OH)^4−^	93.62	3.86	14.93	8.96	− 2.82	80.45	96.49	17
Al_2_(OH)_2_CO_3_^2+^	0.13	0.07	0.00			0.08	0.18	2
Ba^2+^	90.56	2.70	7.28	0.36	− 0.69	84.02	94.72	26
BaCl^+^	0.10	0.05	0.00	− 0.27	0.75	0.03	0.20	26
BaSO_4_ (aq)	5.30	2.25	5.07	1.40	1.09	1.67	11.43	26
BaNO_3_^+^	0.30	0.30	0.09	6.84	2.58	0.05	1.20	14
BaCO_3_ (aq)	0.26	0.09	0.01	2.79	1.21	0.07	0.54	26
BaHCO_3_^+^	3.63	0.94	0.88	1.40	0.96	2.24	6.24	26
FeOH^2+^	0.03	0.02	0.00	5.55	2.22	0.01	0.09	26
Fe(OH)_2_^+^	97.35	1.19	1.42	− 0.69	− 0.36	95.08	99.40	26
Fe(OH)_3_ (aq)	1.49	0.50	0.25	− 0.50	− 0.10	0.43	2.32	26
Fe(OH)_4_^−^	1.14	0.71	0.50	− 0.62	0.61	0.08	2.58	26
Mn^2+^	70.75	7.81	60.93	6.19	1.45	55.41	98.55	26
MnCO_3_ (aq)	18.39	6.49	42.07	2.33	− 0.46	0.14	33.11	26
MnOH^+^	0.07	0.17	0.03	25.67	5.05	0.01	0.90	26
MnCl^+^	0.08	0.04	0.00	− 0.05	0.79	0.02	0.17	26
MnSO_4_ (aq)	4.93	2.32	5.38	2.02	0.69	0.03	11.57	26
MnNO_3_^+^	0.07	0.08	0.01	4.95	2.30	0.01	0.29	13
MnHCO_3_^+^	5.74	1.74	3.02	2.76	− 0.77	0.37	9.29	26
Sr^2+^	86.26	3.59	12.86	− 0.11	− 0.50	78.58	92.02	26
SrCl^+^	0.15	0.07	0.00	− 0.38	0.71	0.05	0.30	26
SrSO_4_ (aq)	7.32	3.01	9.04	1.21	1.01	2.38	15.33	26
SrNO_3_^+^	0.22	0.22	0.05	6.57	2.54	0.04	0.88	14
SrCO_3_ (aq)	0.30	0.11	0.01	2.67	1.17	0.08	0.64	26
SrHCO_3_^+^	5.84	1.46	2.14	1.39	0.95	3.68	9.95	26
Zn^2+^	63.42	6.13	37.53	1.38	− 0.63	49.04	75.59	18
Zn(CO_3_)_2_^2−^	0.14	0.10	0.01	3.30	1.79	0.01	0.42	18
ZnOH^+^	1.25	0.51	0.26	− 0.06	0.31	0.42	2.32	18
Zn(OH)_2_ (aq)	0.56	0.37	0.13	0.20	0.67	0.05	1.40	18
ZnCl^+^	0.21	0.11	0.01	− 0.20	0.82	0.07	0.43	18
ZnSO_4_ (aq)	6.22	2.79	7.77	0.72	0.80	1.73	13.01	18
Zn(SO_4_)_2_^2−^	0.13	0.14	0.02	2.60	1.75	0.01	0.52	17
ZnNO_3_^+^	0.12	0.14	0.02	2.33	1.83	0.02	0.42	9
ZnCO_3_ (aq)	19.57	5.82	33.89	1.91	0.37	6.74	33.64	18
ZnHCO_3_^+^	8.45	2.13	4.54	0.09	0.70	5.31	13.25	18
Cr^2+^	0.98	0.44	0.19	− 2.76	− 0.38	0.45	1.41	4
CrOH^+^	99.02	0.44	0.19	− 2.76	0.38	98.59	99.55	4
V^2+^	16.72	0.90	0.81			16.08	17.36	2
VOH^+^	83.28	0.90	0.81			82.64	83.92	2

A monitoring program is recommended for groundwater quality monitoring to ensure and inhibit water contamination, as mitigation action step toward imminent trace metal poisoning.

### Reduction/oxidation processes and their influence on mitigation actions

Redox conditions play an important role in several geochemical processes that affect groundwater quality such as ion exchange, degradation, sorption, complexation, and mineral dissolution/precipitation (Böhlke, 2002; McMahon et al., 2019; Welch et al., 2000). Those processes have a direct effect on influencing the toxicity, mobility, and transport of anthropogenic contaminants (e.g., arsenic, manganese, and nitrate).

Although Iron is one of most survival vital elements for growth of all living organisms, if it exceeds permissible limits, it greatly affects the benthic invertebrates and fish diversity in canal causing hindering the respiration of fishes (Vuori, 1995; EPA, 2016). Also, aquatic plants as rice will be affected by iron toxicity as results of irrigation with this water leading to leaves acropetal translocation, also bronzing of rice leaves and yield loss (Becker & Asch, 2005; Phippen et al., 2008).

Unlike Iron, Aluminum has no biological role and a toxic nonessential metal to microorganisms. It inhibits the functions of some enzymes since it has a greater affinity to DNA and RNA. It affects the living organism metabolic pathways that involve iron metabolism, phosphorous, fluorine, and calcium, (Olaniran et al., 2013).

### Recommendation toward mitigation actions

Groundwater is vital and critical source for all kind of life species (humans, animals, and plants) especially in arid and semiarid regions of developing countries. The results of this study indicated that the majority of water samples in the study region posing some adverse health consequences for humans in case of using it in drinking or domestic uses. Therefore, some mitigation action should be recommended as follows:

- Minimizing or avoiding the direct spilling of industrial, agricultural, and domestic wastes in water ways as canals or drains, hence there is a direct infiltration to the groundwater system. This can be done proper treatment of hazardous waste and recycling it.
- Adapting new practices in agriculture as organic farming and integrated pest management for decreasing the use of fertilizers in the study area.
- Raising the awareness of the residents for protecting the water sources should be carried out through governments and non-governmental organizations.
- A monitoring program is recommended to ensure and inhibit water contamination, as mitigation action step toward imminent trace metal poisoning and as a step toward the improving of the groundwater quality.

## Conclusion

Groundwater is vital resource as being a life-sustaining factor influences the health of many ecosystems. This study focused on the relationship between sewage treatment plant and groundwater system surrounding it using multidisciplinary approach that combines the characterization of groundwater system and its connection with surrounding canal and drains using chemical and isotopic characterization. In addition, the multivariate statistical analysis has been utilized as a trial to correlate and relate the variables within the whole samples domain by choosing the most threatened variable (physicochemical, chemical, microbial parameters) to elucidate the causes of groundwater pollution in the study area. The potentially toxic trace elements exposure for adults as general group through the direct ingestion and dermal contact exposure pathways were estimated. Visual MINTAQ geochemical modeling program was used for determining the groundwater aqueous species distribution to indicate their effect on the distribution of trace element in groundwater, revealing that Ba, Mn, Zn, and Sr existed as free radical ion in the hazardous samples; illustrating that those elements would be matigated through sorption processes.

The results indicated that the Nile system is the main source of recharge in the groundwater system. Water quality index (WQI) was calculated to assess the drinking water quality in the study area reveling that 58% of the samples are unsuitable for drinking. The contamination consequences on human health were determined through and chronic non-carcinogenic health risk for adults via exposure of both oral and dermal routes. The positive correlation between TDS and Trace elements indicate the continuous dissolution of aquifer matrices minerals and amorphous phases, which was confirmed from Visual MINTAQ geochemical modeling program; continuous dissolution of minerals within the aquifer matrices, except Gibbsite and Barite that was supersaturated. The metal speciation studies of Ba, Mn, Zn, and Sr for the samples existed as free radical ion, and small extent of these elements existed as inorganic ligands in the hazardous samples suggests that those elements would be available for further sorption process and can be mitigated through sorption processes. The mobility of those elements will not increase in our case as results of absence of acidic conditions and low dissolved organic matter content.

## Data Availability

All data and materials as well as software application or custom code support our published claims and comply with field standards.
